# Late Prosthetic Shoulder Hemiarthroplasty after Failed Management of Complex Proximal Humeral Fractures

**DOI:** 10.1155/2013/403580

**Published:** 2013-01-09

**Authors:** A. Panagopoulos, P. Tsoumpos, K. Evangelou, Christos Georgiou, I. Triantafillopoulos

**Affiliations:** ^1^Department of Shoulder & Elbow Surgery, Orthopaedic Clinic, University Hospital of Patras, Papanikolaou 1, 26504 Patras, Greece; ^2^Department of Shoulder & Elbow Surgery, Metropolitan Hospital Athens, Medical School, University of Athens, Ethnarxou Makariou & El. Benizelou 1, N. Faliro, 18547 Piraeus, Greece

## Abstract

*Background*. The purpose of this study was to report our experience with shoulder hemiarthroplasty in the context of old trauma. *Methods*. 33 patients with failed treatment for a complex proximal humeral fracture underwent prosthetic hemiarthroplasty. There were 15 men and 18 women with a mean age of 58.1 years. The average period from initial treatment was 14.9 months. Sequelae included 11 malunions, 4 nonunions, 15 cases with avascular necrosis (AVN) and 3 neglected posterior locked dislocations. Follow up investigation included radiological assessment and clinical evaluation using the Constant score and a visual analogue pain scale. *Results*. After a mean follow up of 82.5 months the median Constant score was 75.7 points, improved by 60% in comparison to preoperative values. Greater tuberosity displacement, large cuff tears and severe malunion were the factors most affected outcome. No cases of stem loosening or severe migration were noted. 60% of the patients were able to do activities up to shoulder level compared with 24% before reconstruction. *Conclusions*. Late shoulder hemiarthroplasty is technically difficult and the results are inferior to those reported for acute humeral head replacement, nonetheless remains a satisfactory reconstructive option when primary treatment fails.

## 1. Introduction

Shoulder hemiarthroplasty is a technically challenging procedure which can predictably restore shoulder-level function in patients with 4-part fractures, some 3-part fractures, fracture dislocations, head-splitting fractures, and impaction fractures of the humeral head with involvement of more than 50% of the articular surface [1–4]. Early surgical intervention within 2 weeks postinjury, accurate tuberosity reconstruction, and appropriate height and retroversion of the prosthesis are the factors with the greatest impact on functional outcome [5–8]. 

In contrast, outcomes of internal fixation [9, 10] and nonoperative treatment [11, 12] for these complex fractures are quite controversial, with the initial management considered critically important. Krappinger et al. [13] showed in a recent study that multifragmentary fracture patterns in old patients with low local BMD are prone for fixation failure. Revision osteosynthesis or late prosthetic shoulder arthroplasty in these complex fractures is fraught with complications, and functional results are usually disappointing [14, 15]. Bone loss, malunion, ectopic ossification, avascular necrosis, associated rotator cuff tears, and severe contractions of soft tissues are some of the factors that prevent appropriate prosthesis placement and postoperative rehabilitation, thus currently a reverse shoulder arthroplasty is considered as the treatment of choice for these injuries. 

The aim of this study is to present the long-term outcome of 33 late prosthetic shoulder replacements carried out on patients who had failed conservative or operative treatment for complex fractures of the proximal humerus.

## 2. Materials and Methods

Between 2004 and 2007, thirty-eight patients underwent shoulder hemiarthroplasty after failed conservative or operative treatment for complex proximal humeral fractures in our department. Three patients were lost from followup, and two died from reasons unrelated to the fracture leaving a cohort of 33 patients, with a minimum followup of 5 years, for the outcome analysis. There were 18 women and 15 men with a mean age of 58.1 years old (range, 34 to 83 years old) at implantation. The dominant arm was involved in 23 (69.7%) cases. Seven patients performed heavy or manual labor, fifteen were sedentary, and eleven were retired.

The type of initial fracture according to Neer classification [5] was a 2-part surgical neck fracture in 2 patients, a 3-part fracture in 5, a 4-part fracture in 12, a 3- or 4-part fracture dislocation in 7, and a neglected “locked” posterior fracture dislocation in 3. Four patients who have been referred to us by other hospitals had no immediate postinjury radiographs, and the type of fracture could not be identified. The initial treatment was conservative in 16 patients and surgical in 17. In the open group, 6 patients had been managed with transosseous suturing fixation [16] (all in our department), 8 patients with plate-screw osteosynthesis, and 2 patients with screw-wiring osteosynthesis (Table 1). Additional operations prior to hemiarthroplasty have been performed in 5 patients: a plate-screws exchanged osteosynthesis after suturing fixation due to nonunion, an open release of the shoulder joint due to adhesive capsulitis after suturing fixation, a hardware removal, and surgical debridement after a persistent deep infection and a McLaughlin procedure for a neglected “locked” posterior dislocation. Another case underwent hardware removal in another center prior to final referral. 

**Figure 1:**
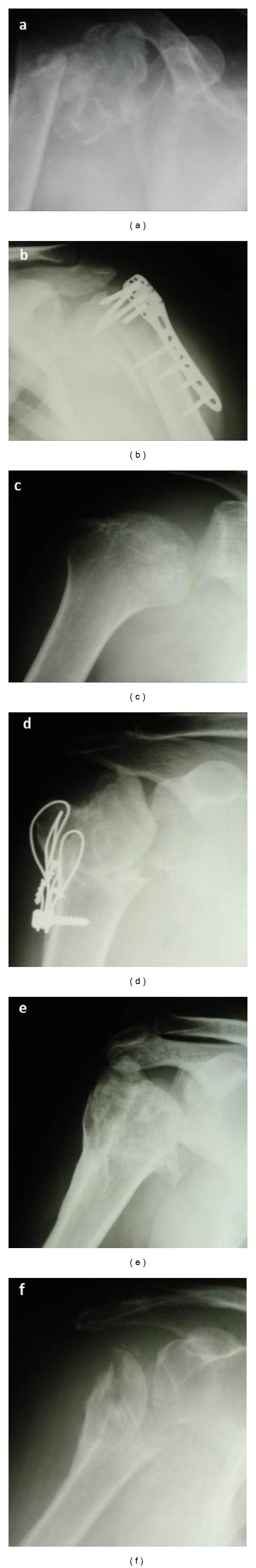
Types of sequelae of proximal humeral fractures after initial treatment. (a) Conservative, (b) plate osteosynthesi, (c) neglected locked posterior dislocation, (d) screw-wiring osteosynthesis, and (e) and (f) transosseous suturing [16].

With the exception of the “locked” posterior dislocations, the main complication of initial treatment was malunion in eleven cases, nonunion in four, avascular necrosis (AVN) in fourteen, and septic AVN in one but without evidence of active infection (Figure 1). All patients underwent prosthetic hemiarthroplasty in an averaged delay period from the original injury of 12.7 months (range, 2 to 32 months).

Prior to arthroplasty and at the last followup appointment, subjective pain and overall function were evaluated with a visual analog scale (from 0 (maximum pain) to 10 (no pain at all)) and according to the parameters of Constant-Murley score, respectively, Pain, performance of daily activities, range of motion, and strength were scored on a scale of 1 to 100, with 100 being an excellent score.The isometric power of the shoulder was assessed by assigning a maximum of 25 points when a patient could resist a maximal weight of 12 kg at 90° of shoulder abduction or when the resisted weight was lesser but equal to the resisted weight on the contralateral noninjured arm. 

An excellent or very good result was considered if the patient expressed none or little pain, reported normal use of his arm and had an objective improvement of shoulder function by at least 75% or 50%, respectively, when these values were compared with the preoperative ones. Unsatisfactory results comprised those cases with moderate or severe pain and objective improvement of shoulder function less than 25% in contrast to the preoperative values. Patients who had values between these limits (25%–50%) were considered as having a moderate outcome. Clinical evaluation was performed by two independent with the project observers (PT and KE). Finally, all patients were asked about their satisfaction with the final result and if they were agreed to undergo the procedure again under similar circumstances.

Radiological evaluation was performed with standardized “trauma series” views of good quality both preoperatively and postoperatively (Figure 1). Additional CT scans with three-dimensional reconstruction were performed in 18/33 patients for further assessment of malunion, articular incongruence, and bone stock quality. A ^99m^Tc-bone scan was performed in one patient to exclude the presence of active infection. The most recent radiographs were reviewed by a senior of us (I.T.) not involved in the initial treatment to determine the presence of periprosthetic loosening and heterotopic ossification and to evaluate the stem properties and greater tuberosity position.

## 3. Surgical Technique

Our surgical technique was similar to that original described by Neer [5, 17]. As there were not any preoperative indications or intraoperative findings of severe glenoid degeneration, all patients were managed with shoulder hemiarthroplasty without glenoid replacement. Two types of prosthetic implants were used (Neer II and Biomet). The deltopectoral approach was used in all cases. In 9 cases, diffuse adhesive capsulitis was noted requiring extended capsular release for achieving a functional range of motion. Full-thickness rotator cuff tears were noted in 4 cases (2 of supraspinatus tendon and two of both supraspinatus and infraspinatus tendons), whereas partial-thickness tears was detected in 7 cases; all tears were repaired with non-absorbable Ethibond-2 sutures. The biceps tendon was normal in 20 cases, frayed or degenerative in 11, and complete ruptured in 2 cases. Biceps tenodesis was performed in 8 cases. Greater tuberosity osteotomy was performed in 7 cases and double osteotomy of both tuberosities in 3 cases. Two to three pairs of heavy nonabsorbable sutures (Ethibond no.5) were applied to each tuberosity near the insertion of the adjacent tendon prior to removal of the humeral head. The later was carefully showed off in its anatomical neck, and the humeral canal was prepared without aggressive reaming. Two 2.7 mm drill-holes were created in each side of the diaphysis both laterally and medially, and two pair of sutures were placed for tuberosity fixation. Humeral component was placed after cementing the canal at the appropriate height, with 30°–35° of retroversion. Tuberosity fixation was performed thereafter with the horizontal intertuberosity sutures incorporated to the lateral fins of the prosthesis and the vertical diaphyseal tuberosity sutures in a cruciate tension band fashion that ensures stable fixation of the construct and adequate balance of the adjacent rotator cuff tendons. Additional cancellous bone grafting was placed into the proximal humeral space in 6 cases for filling the spaces between the implant and the tuberosities. Finally, the rotator cuff interval was closed with separate sutures and the deltopectoral space with absorbable sutures in a figure of eight manner. The arm was rested in internal rotation in a simple sling with the elbow at the side for 4–6 weeks or was immobilized in abduction in a special brace if a full-thickness rotator cuff or tuberosity osteotomy had been performed. 

A closely monitored 3-phase rehabilitation program given to all patients initially consisted of pendulum exercises starting on the 2nd postoperative day until the 3rd to 4th postoperative week. The second phase includes passive assisted exercises in the supine position as the patient is trying to reach the bed, supporting his injured shoulder by the healthy arm or special designed sticks. Until the 6th to the 7th postoperative week, forward elevation and external rotation are performed in the supine position while internal rotation in the standing one with the aid of sticks. As the union of the tuberosities is completed, active exercises using gradually increased weights (starting from 1 kg) are administered until the 10th to the 12th postoperative week. If the patient was capable of forward elevating three kilos in the supine position, active dynamic shoulder motion, and strengthening exercises are administered in the standing position until the 5th to the 6th postoperative month. Preservation of shoulder motion and strength is maintained for another 3 to 4 months. The patient is seen every single week for the first 2 to 3 postoperative months and is instructed and guided by us. We believe that a simple prescription of physiotherapy does not help the patient as much as this close and monitoring consultation with his surgeon. 

## 4. Results 

**Figure 2:**
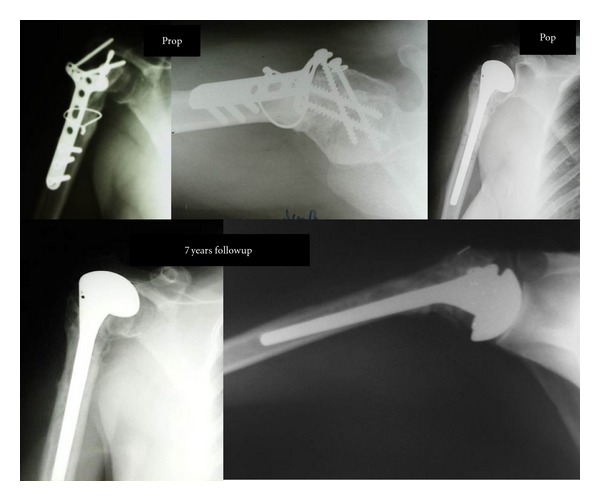
Patient no. 31: hemiarthroplasty after failed internal fixation with plate-screws osteosynthesis. Very good radiological and clinical result (Constant score = 85) seven years postoperatively.

**Figure 3:**
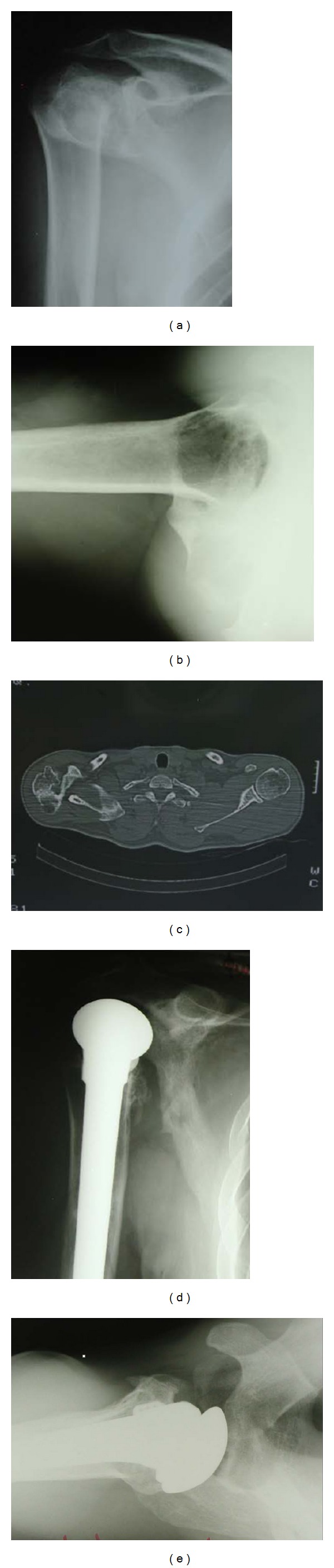
Patient no. 30: neglected posterior locked dislocation treated conservatively elsewhere. Very good result 5.5 years postoperatively with a Constant score of 76 points.

**Figure 4:**
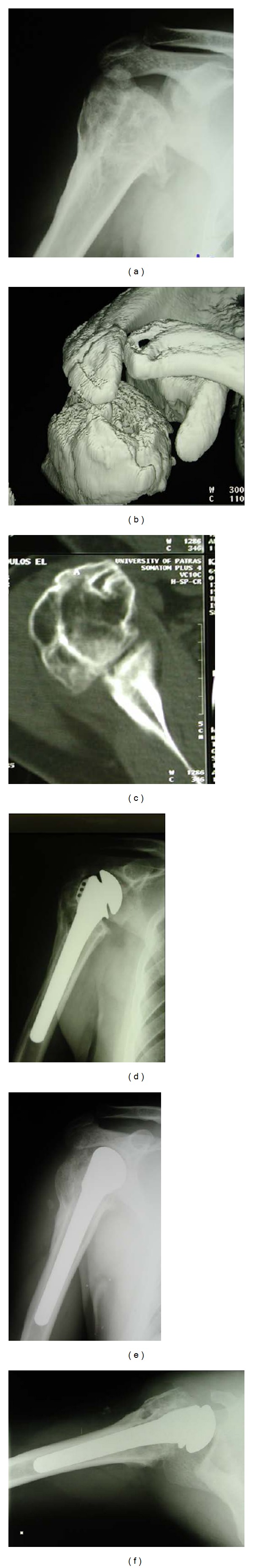
Patient no. 1: AVN of the humeral head after trasosseous suturing fixation for a 4-part valgus impacted fracture initially managed to our department. Anteroposterior radiographs in external and internal rotation showed excellent tuberosity healing 7 years postoperatively, although there were slight upward migration of the prosthesis and eccentric position in the axillary view. Despite that, the patient was clinically pain free with a Constant score of 80 points.

The mean followup period was 82.5 months (range, 61 to 96 months). The median Constant score was 75.7 points, improved by 60% in comparison to preoperative values (mean, 47.9 points). In the pain analogue scale, there was an averaged improvement from 4 to 8 points. According to our criteria, the result was excellent in nine patients, very good in eleven, moderate in ten and unsatisfactory in four. Active forward elevation increased from 56 degrees to 100 degrees, active external rotation increased from 12 degrees to 35 degrees, and finally, active internal rotation increased from the ability of the thumb to reach the sacrum (range, greater trochanter to the first lumbar vertebral body) to the second lumbar vertebral body (range, trochanter to T7). Sixty per cent of the patients were able to do activities up to shoulder level compared with 24% before arthroplasty (Figure 2). Overall, 79% of the patients were satisfied with the final outcome and said that they would repeat the operation in similar circumstances (Figures 2, 3, and 4).

Periprosthetic ossification was present in 7 cases. It was minimal in 4 cases, predominant at the humeral side in 2, and near the glenoid in 1. All the humeral implants were normally positioned, except two that were placed in valgus orientation. Slight upward positioning of the implant was noted in three cases and partial anterior subluxation in two. Early loss of greater tuberosity fixation or GT nonunion was not noted, but osteolysis was detected in 3 cases and malunion in two who expressed an unsatisfactory outcome. Four humeral components had radiolucent lines without evidence of loosening. 

## 5. Discussion

Complications after proximal humeral fracture fixation are some of the most difficult situations to manage in shoulder reconstruction. An anticipated and reliable functional result is difficult to obtain because of the complexity of bone pathology and the impaired soft tissue envelope, especially if the patient had already undergone surgery. The surgeon has to deal with malunion, nonunion or AVN of the proximal humerus, displacement of the tuberosities, rotator cuff tears, and associated soft-tissue contractures. In the presence of sever osteoporosis, significant bone loss, articular incongruity, and glenoid erosion, the only indication is prosthetic replacement of the proximal humerus. Generally, a satisfactory result may be expected in 20% to 75% of the cases, with pain relief obtained in more than 85% [6, 18, 19]. Kontakis et al. [3] in a systematic review of 810 early hemiarthroplasties in 808 patients for proximal humeral fractures not only concluded that most patients had no pain or only mild pain but also that the level of function before injury was almost never regained. In the present study of late hemiarthroplasties for proximal humeral fractures, 29/33 patients had a good or acceptable, even moderate outcome, whereas improvement of pain was up to 80%. 

The results of shoulder replacement for old trauma are much less favorable than those of primary osteoarthritis or hemiarthroplasty performed for acute fractures [6, 20, 21]. Fevang et al. [22] in a large series of 1.825 shoulder arthroplasties of the Norwegian Registry found that the risk of revision was the highest for patients with sequelae after fracture compared to those with acute fractures. Several other factors have been proposed to alter final outcome such as the age of the patient, initial treatment (conservative or surgical), the type of the sequelae (malunion, nonunion, AVN, and glenoid erosion), the need for tuberosity osteotomy, associated rotator cuff tears, and the condition of soft tissues. 

In contrast to similar reports [16, 22, 23], the age of the patient did not influence the final outcome in the present study. Bosch et al. [24] stated that what seems to be more important for rehabilitation is the cooperation and mental status of patients, rather than their age. If the patient is closed monitored and instructed by his surgeon the results are more predictable, because the physiotherapy can be focused to the most impaired function.

Norris et al. [14] emphasized the crucial role of the initial fracture treatment for the final results of arthroplasty; the patients who had been managed conservatively had a better result than those who have been operated. We did not notice any difference in our study regarding the initial treatment. From the 17 cases that had been treated operatively, six were managed solely with transosseous sutures, and the consequences of sequelae of the proximal humerus were minimal. Accordingly, these patients were closed monitored as they had received initial treatment in our department, and a good rate of shoulder motion had been achieved prior to arthroplasty; four of them had signs of AVN, and their main complain was pain and not restriction of motion. On the other hand, most of the patients that had been managed in other centers with metallic internal fixation referred to us also for their pain due to AVN or nonunion. Finally, most of the patients that had been treated conservatively elsewhere showed moderate or severe malunion, but as they referred to us early (2-3 months later), the quality of bone, soft tissues, and rotator cuff tendons were less disturbed. 

The type of complication after failed initial treatment of a proximal humerus fracture is considered as a crucial factor for the final outcome [6, 20, 25]. Dines et al. [15] reported better results in fractures with AVN in comparison to nonunited or malunited fractures. The present study showed that the results were slightly better in fractures that had complicated mainly with AVN, but the difference was not important. A satisfactory outcome was noted also in all the three cases with neglected posterior “locked” fracture dislocations. The average functional improvement was up to 84%, a result similar to Neer [17] who reported excellent results in 76% of the patients with postdislocation arthropathy. 

Glenoid replacement is another predisposing factor of the final outcome [26, 27]. Dines et al. [15] in a recent study of modular prosthesis showed a better result for hemiarthroplasty, in contrast to total shoulder replacement. The survivorship analysis of Fevang et al. [22] showed that for hemiprostheses, the major cause of revision was pain, seen in 15 of 439 cases with rheumatoid arthritis but in none of the 422 cases with acute fractures. None of our patients received total shoulder replacement, although mild erosion of the glenoid was detected in two of them. We do not suggest glenoid replacement even in cases with slight abnormal cartilage degeneration, especially when the condition of soft tissues and rotator cuff tendons are severely disturbed. 

Finally, the negative effect of tuberosity osteotomy and subsequent malunion and/or nonunion in prosthetic replacement of the shoulder has been suggested by many authors [6–8, 21, 24, 28]. Neer [17] has already suggested that in borderline malunions it is better to use prosthesis with a small stem and a small head, in a varus position, to avoid having to perform a greater tuberosity osteotomy. Franta et al. [29] in a multifactorial analysis of 282 unsatisfactory arthroplasties reported that patients with a proximal humerus nonunion were at 20 times greater risk for tuberosity failure than all other diagnoses. In addition, tuberosity failure was found to be significantly associated with humeral component loosening. In the present study, GT osteotomy was performed in 7/33 cases, but only two patients had unsatisfactory results. The amount of GT osteotomy and mainly the type and adequacy of fixation seem to influence the final outcome.

Our study has several limitations: the number of involved patients is quite small, and although have been managed similarly they represent a mixed group of various sequelae of proximal humeral fractures including both conservative and operative treated cases. One can expect better results in the first group as well as in those patients treated with head preserving surgery, but this was not true in our study and statistical differences could not be established. Furthermore, the data were derived from only one practice with great experience in shoulder reconstruction and, as such, may not be generalizable to all practices. 

## 6. Conclusion

Shoulder hemiarthroplasty for management of posttraumatic complications of fracture of the proximal humerus is a technically demanding procedure with unpredictable results. The high rate of complications is often related to technical difficulties, a scarred deltoid, adhesions of rotator cuff tendons, and malunion of the tuberosities. Careful selection of the patients, detail preoperative planning, and meticulous surgical technique are essential elements for a successful outcome. The postoperative rehabilitation program should be modified based on the surgical findings and the technique used. In this manner, certain possible secondary complications could be avoided, and the long-term results will be more favorable.

## Figures and Tables

**Table 1:** Overview of clinical data.

Patient name	Age, gender	Side	Type of fracture	Initial treatment or reoperation	Time from injury to arthroplasty/m	Type of sequelae operative findings	Followup (months)	Constant score		Pain scale	
								Preop	Fup (*)	Preop	Fup
(1) KE	38, m	R	4-part	SF, capsular release	36	AVN, M, RCT	84	55	80 (45%)	4	9
(2) TH	62, m	R	3-part	SF, PLO	12	N, RCT, GTO, LTO	92	47	80 (70%)	5	10
(3) SB	67, f	R	2-part surg. neck	SF	7	AVN	90	45	80 (70%)	3	9
(4) LX	72, f	R	3-part disloc.	PLO, HDR (elsewhere)	18	N, EO, BL	96	48	80 (66%)	2	10
(5) KI	49, m	R	No specified	SWO (elsewhere)	7	AVN, M, HMF, GTO	72	56	90 (60.7%)	5	10
(6) TP	47, f	R	No specified	PLO (elsewhere)	28	AVN, M, HMF	65	55	81 (47%)	4	9
(7) MH	53, m	R	4-part fr/dis	C (elsewhere)	2	M, EO, GTO	70	45	65 (44%)	5	6
(8) XE	72, f	L	4-part fr/dis	SF	13	M, RCT	92	55	75 (36%)	4	8
(9) KM	77, f	L	4-part fr/dis	C (elsewhere)	4	M, EO,	90	43	86 (100%)	5	9
(10) MB	70, f	R	4-part	C (elsewhere)	32	AVN, EO	91	47	85 (80%)	4	10
(11) LB	67, f	L	No specified	SWO + IMW (elsewhere)	32	AVN, M, GTO, LTO	86	45	85 (88%)	2	8
(12) DX	50, f	L	No specified	PLO (elsewhere)	5	M, HMF, BL	92	35	83 (100%)	3	9
(13) AF	35, m	R	4-part	C (elsewhere)	2	AVN	75	52	87 (67%)	5	10
(14) PE	61, f	R	4-part disloc.	C (elsewhere)	4	AVN	65	54	79 (46%)	3	7
(15) BN	34, m	R	4-part	C (elsewhere)	2	AVN, M	61	51	82 (40%)	4	8
(16) ZS	83, m	R	3-part disloc	C (elsewhere)	2	M, GTO, LTO	80	43	70 (62%)	3	9
(17) DM	68, f	L	4-part disloc.	C (elsewhere)	3	M, AVN, EO	89	44	78 (77%)	3	7
(18) TE	62, f	R	2-part surg. neck	C (elsewhere)	4	M, EO	85	52	76 (46%)	4	8
(19) TB	65, m	L	3-part	C (elsewhere)	12	M, AVN, GTO	92	54	65 (20%)	5	9
(20) KE	62, f	L	4-part	PLO, infection, HDR	40	AVN, M, RCT	85	47	55 (17%)	4	3
(21) AB	59, m	R	4-part	C (elsewhere)	6	M, AVN, BL, EO	77	53	75 (41%)	5	10
(22) KX	48, m	R	PLFD, neglected	McLaughlin procedure	24	D, RCT, block of rotation	96	38	70 (84%)	5	8
(23) FT	34, m	R	PLFD, neglected	C (elsewhere)	15	D, block of rotation	89	47	85 (80%)	5	8
(24) PE	67, f	L	3-part	C (elsewhere)	12	M, AVN, GTO	88	30	32 (6%)	4	0
(25) KM	59, m	R	4-part	SF	21	AVN	64	43	54 (25%)	5	5
(26) AA	70, f	R	4-part	PLO (elsewhere)	2	M, EO	72	47	82 (75%)	4	8
(27) DB	61, f	L	3-part	C (elsewhere)	7	N, EO	91	49	80 (63%)	5	9
(28) TA	46, m	R	4-part	SF	14	AVN	80	52	82 (57%)	4	9
(29) MX	44, f	L	4-part	PLO (elsewhere)	8	N, AVN, EO	80	50	77 (54%)	3	7
(30) KP	62, m	R	PLFD, neglected	C (elsewhere)	13	D, block of rotation	67	42	76 (80%)	5	8
(31) TE	49, m	L	4-part	PLO	11	AVN	84	53	85 (60%)	5	8
(32) DS	60, f	R	4-part	C (elsewhere)	3	AVN, M	90	52	67 (29%)	3	6
(33) KS	65, f	R	3-part	PLO	18	AVN	91	54	74 (37%)	4	8

C: conservative treatment, D: dislocation, SF: suturing fixation, PLO: plate-osteosynthesis, M: malunion, N: nonunion, AVN: avascular necrosis, RCT: rotator cuff tear, EO: ectopic ossification, GTO: greater tuberosity osteotomy, LTO: lesser tuberosity osteotomy, HDR: hardware removal, BL: bone loss, SWO: screw-wiring osteosynthesis, HMF: hard material failure, IMW: intramedullary wiring, and PLFD: posterior “locked” fracture dislocation.
